# Response to: Comment on: “After Dinner Rest a While, After Supper Walk a Mile? A Systematic Review with Meta-analysis on the Acute Postprandial Response to Exercise Before and After Meal Ingestion in Healthy Subjects and Patients with Impaired Glucose Tolerance”

**DOI:** 10.1007/s40279-023-01883-4

**Published:** 2023-07-22

**Authors:** Tobias Engeroff, David A. Groneberg, Jan Wilke

**Affiliations:** 1 Division Health and Performance, Institute of Occupational-, Social- and Environmental Medicine, Goethe-University Frankfurt, Theodor-Stern-Kai 7, Building 9B, 60590 Frankfurt am Main, Germany; 2 Department of Movement Sciences, Institute of Sport Sciences, University Klagenfurt, Klagenfurt, Austria

Dear Editor,

We appreciate the Letter to the Editor by Elsamma Chacko [1], which adds valuable information and puts the results of our review and meta-analysis [2] in a clinical context. Based on our analysis [2] and earlier work on postprandial and postresorptive glucose [3, 4] and lipid metabolism [5] during physical activity, we agree with Dr. Chacko’s [1] assumptions concerning one of the key mechanisms behind the beneficial effects of postprandial exercise on glucose and insulin metabolism. During exercise in the fasted state, the body uses energy reserves, mainly in the form of glycogen from muscle and liver combined with fatty acids from various sources. Contrastingly, the orally ingested glucose can be used immediately by the working muscles during postprandial exercise. Accordingly, the onset of muscle activity should be coordinated with the occurrence of elevated blood glucose levels, which depends, among other things, on the glycemic load as well as on the duration of food intake.

As mentioned in our discussion [2], the number of included studies is very small, while the variance in time between food intake and exercise, as well as the variance in type and composition of the meals, is very large. Furthermore, only one of the included studies [6] analyzed the effect of different meal-activity timings in a randomized controlled trial (RCT) with multiple postprandial exercise interventions. Despite the limited evidence and the variance in food, our moderator analysis shows agreement with Solomon’s results [6] that starting physical activity after eating is preferable to delaying it for 30 min or longer. Our meta-analytic evaluation seems therefore not consistent with Dr. Chacko’s clinical experience concerning the optimal time point of exercise [1]. We believe that this may be due in part to the small number of studies evaluating effect in participants with impaired glucose tolerance. We therefore reiterate the call for further studies to provide more clarity on the impact and interaction of food composition and meal-exercise timing. As Dr. Chacko correctly points out [1], another question that is not sufficiently clarified is the most appropriate form of activity. In this regard, from our perspective, a systematic analysis of the following exercise prerequisites is needed: energy expenditure, exercise intensity (with regard to the metabolic processes and substrate utilization) and exercise type [especially comparing (a) interval protocols with continuous exercise and (b) analyzing the impact of the muscle groups used]. Furthermore, the effect of repeated exercise bouts [(a) exercise after multiple meals on one day and (b) exercise after the same meal over the time frame of multiple weeks] needs to be further analyzed.

Especially with regard to this lack of evidence and the varying glycemic load of different meals, we therefore support Dr. Chacko’s [1] proposal to test different exercise types and meal–exercise time intervals at an individual level. From our point of view, the shortest interval should be starting with activity immediately after eating.

## Declarations

### Data availability

There are no data/results in this letter.
